# Author Correction: Systemically administered wound-homing peptide accelerates wound healing by modulating syndecan-4 function

**DOI:** 10.1038/s41467-023-44574-4

**Published:** 2024-01-03

**Authors:** Horacio Maldonado, Bryan D. Savage, Harlan R. Barker, Ulrike May, Maria Vähätupa, Rahul K. Badiani, Katarzyna I. Wolanska, Craig M. J. Turner, Toini Pemmari, Tuomo Ketomäki, Stuart Prince, Martin J. Humphries, Erkki Ruoslahti, Mark R. Morgan, Tero A. H. Järvinen

**Affiliations:** 1 Institute of Systems, Molecular & Integrative Biology, University of Liverpool, Liverpool, UK; 2 Faculty of Medicine and Health Technology, Tampere University & Tampere University Hospital, Tampere, Finland; 3 Wellcome Trust Centre for Cell-Matrix Research, University of Manchester, Manchester, UK; 4 Cancer Center, Sanford Burnham Prebys Medical Discovery Institute, La Jolla, CA and Center for Nanomedicine, University of California (UCSB), Santa Barbara, CA USA

**Keywords:** Recombinant peptide therapy, Molecular medicine, Regenerative medicine, Drug delivery

Correction to: *Nature Communications* 10.1038/s41467-023-43848-1, Article published online 06 December 2023

In this article, the author’s name Craig M. J. Turner was incorrectly written as Craig M. L. Turner. The original article has been corrected.

