# Supplementary material for: Systemically administered wound-homing peptide accelerates wound healing by modulating syndecan-4 function
Source: Nat Commun. 2023 Dec 6;14:8069. doi: 10.1038/s41467-023-43848-1 (PMC10700342; doi:10.1038/s41467-023-43848-1)
Supplement: Supplementary file 1 — Supplementary Information [file 41467_2023_43848_MOESM1_ESM.pdf]

**SUPPLEMENTARY INFORMATION**

# Systemically administered wound-homing peptide accelerates wound healing by modulating syndecan-4 function

Horacio Maldonado[1], Bryan D. Savage[1], Harlan R. Barker[2], Ulrike May[2], Maria Vähätupa[2], Rahul K. Badiani[1], Katarzyna I. Wolanska[1], Craig M.L. Turner[1], Toini Pemmari[2], Tuomo Ketomäki[2], Stuart Prince[2], Martin J. Humphries[3], Erkki Ruoslahti[4], Mark R. Morgan[1]*, Tero A.H. Järvinen[2,4]*

## CONTENTS

**Supplementary Figures & Legends**

Supplementary Figure S1

Supplementary Figure S2

Supplementary Figure S3

Supplementary Figure S4

Supplementary Figure S5

Supplementary Figure S6

Supplementary Figure S7

Supplementary Figure S8

**Supplementary References**

**SUPPLEMENTARY FIGURES & LEGENDS**

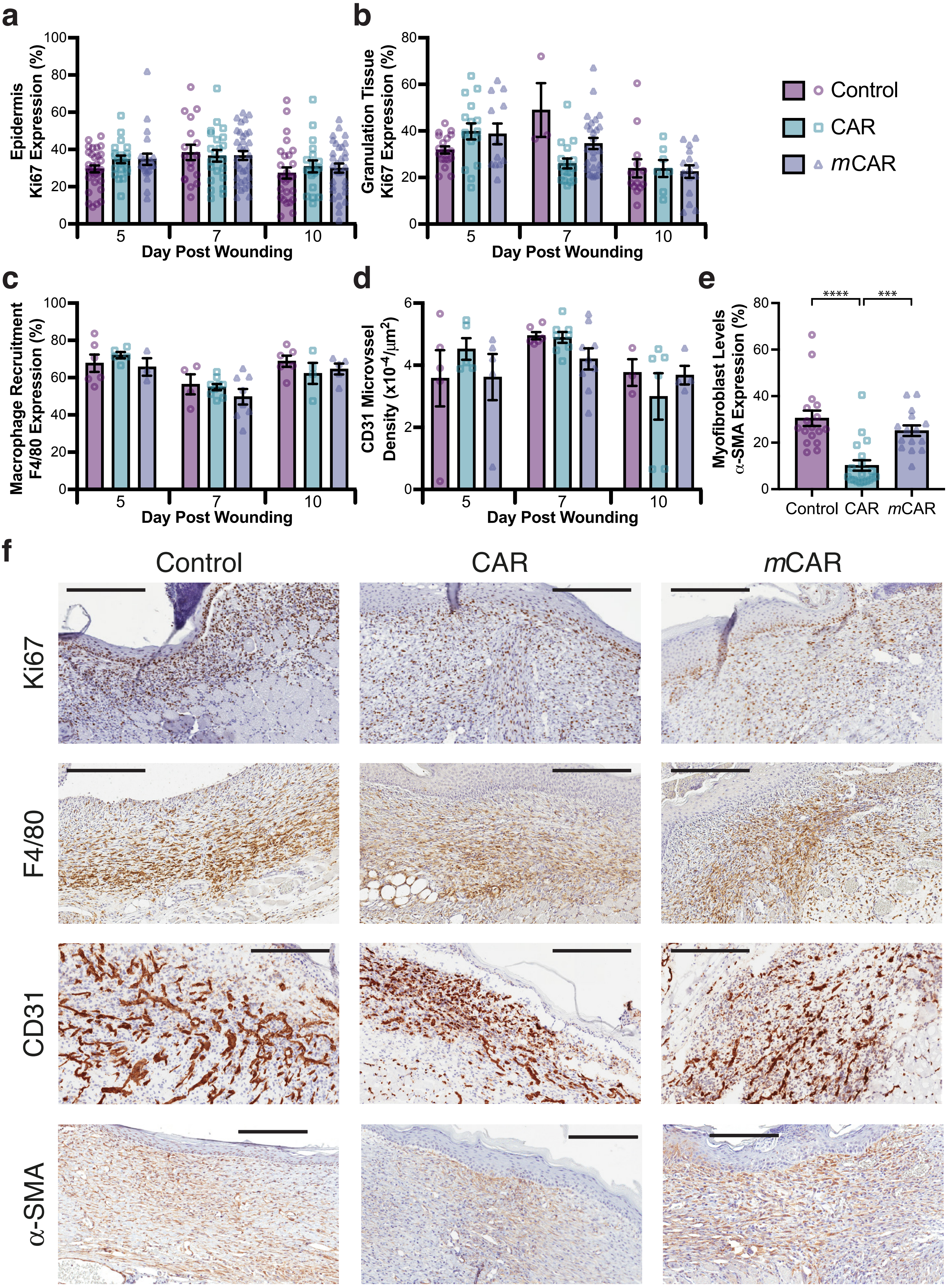
a
Epidermis Ki67 Expression (%)
Day Post Wounding
b
Granulation Tissue Ki67 Expression (%)
Day Post Wounding
Control
CAR
mCAR
c
Macrophage Recruitment F4/80 Expression (%)
Day Post Wounding
d
CD31 Microvssel Density (x10^-4/µm^2)
Day Post Wounding
e
Myofibroblast Levels α-SMA Expression (%)
****
***
Control CAR mCAR
f
Control
CAR
mCAR
Ki67
F4/80
CD31
α-SMA

Figure S1

**Supplementary Figure S1. Cell proliferation, macrophages and angiogenesis are not affected by CAR peptide in skin wounds, but wound contraction takes place without myofibroblasts.** Mice with full thickness skin excision wounds were treated with CAR, *m*CAR and control (BSA/PBS) peptide. Wounds were harvested on days 5, 7 and 10 and were assessed by IHC staining and quantitative image analysis. **(a)** Percentage of proliferating cells stained with Ki67 in the hyperproliferative epidermis and **(b)** in granulation tissue. **(c)** Percentage of F4/80 positive macrophages in the granulation tissue. **(d)** Area covered by CD31-positive blood vessels (microvessel density). **(e)** Percentage of α-SMA-positive myofibroblasts day 10. **(f)** Representative micrographs of skin wound sections stained by IHC for proliferating cells (Ki67) (day 5), macrophages (F4/80) (day 7), blood vessels (CD31) (day 5), and α-SMA positive cells (day 10) are presented for each treatment group. The percentage of positive cells in **a, b, c & e** is shown as percentage of total nucleic cells. Scale bar: 300 µm for Ki67 & CD31; 200 µm for F4/80 & α-SMA. Each data point represents an individual wound. Values are mean ± S.E.M. N = 15 – 19 wounds in each group for α-SMA, N = 7 – 26 for Ki67 in granulation tissue and N = 17 – 57 in epidermis, N = 3 – 9 for F4/80 and N = 3 – 9 for vessel density (CD31). **e)** Control vs CAR P=$2.4x10^{-5}$; Control vs *m*CAR P=0.0005 Kruskal-Wallis rank sum test with Dunn´s test with tie correction. Source data are provided as a Source Data file.

# Figure S2

**a**

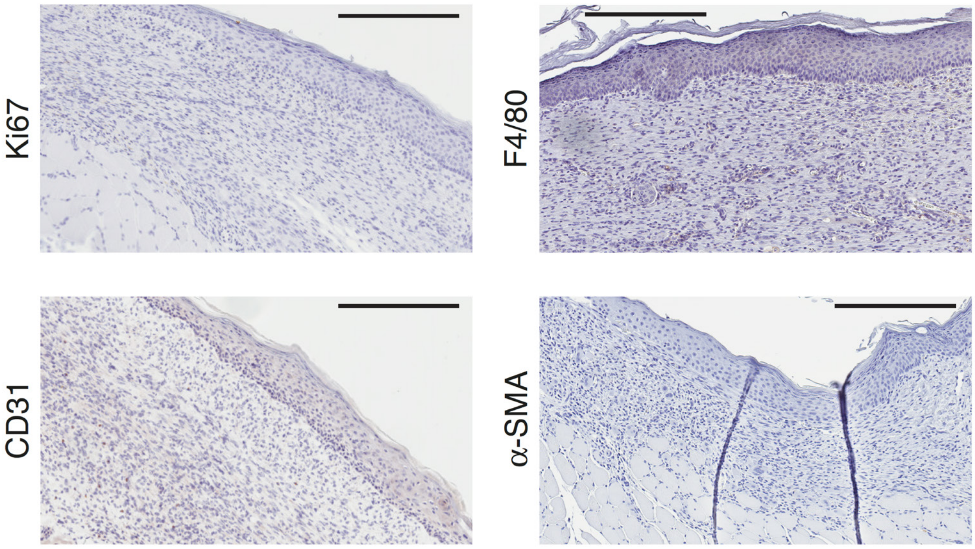

**Supplementary Figure S2. CAR peptide wound treatment: negative controls for IHC stained wound tissue sections from Supplementary Fig. S1**

**(a)** Representative micrographs of skin wound sections stained by IHC with only the secondary antibody as a negative control for the staining of proliferating cells (Ki67), macrophages (F4/80), blood vessels (CD31) and α-SMA positive cells (α-SMA) are shown. Scale bars: 300 µm for Ki67 & CD31; 200 µm for F4/80 &α-SMA.

Figure S3

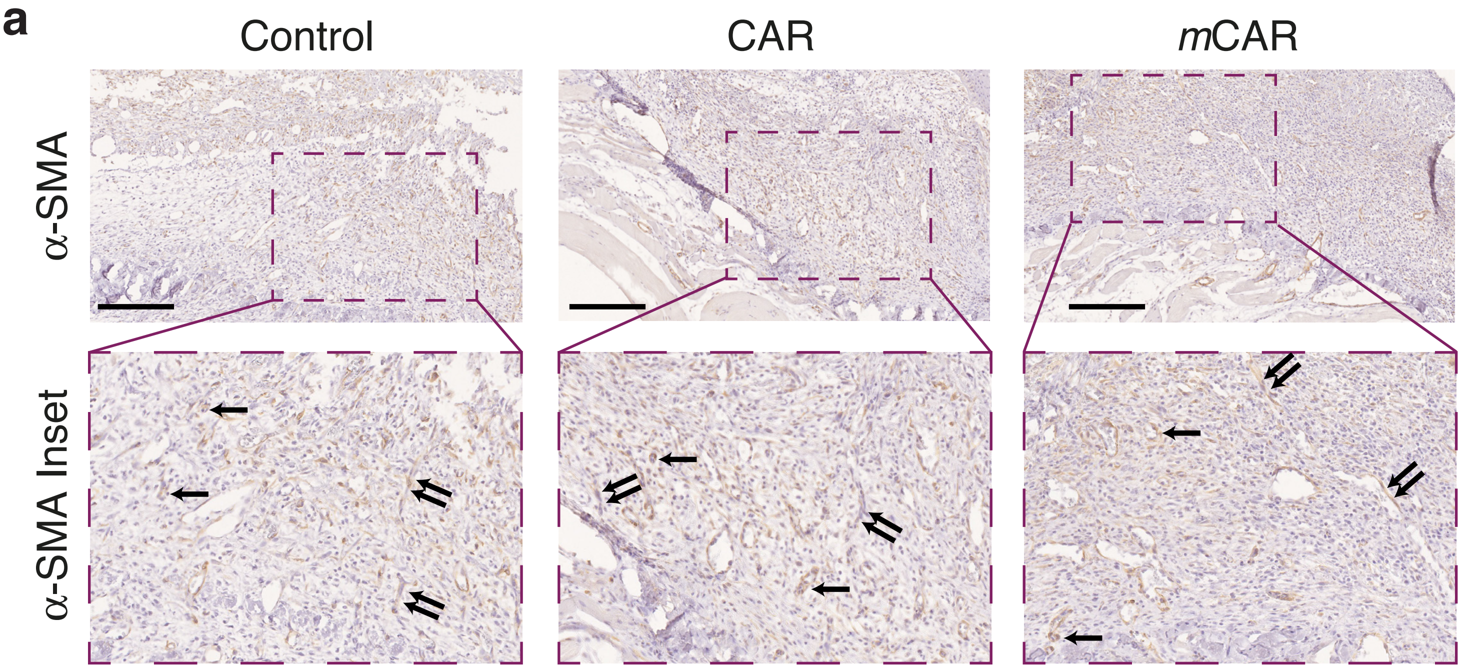

**Supplementary Figure S3. CAR peptide does not induce myofibroblast transformation in early phase of wound healing**

Mice with full thickness skin excision wounds were treated with CAR, *m*CAR and control (BSA/PBS). Wounds were harvested on day 7 and were assessed for myofibroblasts by IHC staining (α-SMA). **(a)** Representative micrographs of skin wound sections stained by IHC for α-SMA positive cells are presented for each treatment group. No myofibroblasts were detected in granulation tissue of day 7 skin wounds in any treatment group. Blood vessel smooth muscle cells exhibit strong α-SMA staining. Scale bar: 250 µm. Dashed box demarcates region of image inset. Of the many α-SMA-positive blood vessels in the granulation tissue, two cross-sectional blood vessels (single horizontal arrow) and two longitudinal blood vessels (double diagonal arrows) are highlighted in each image. Images are directly comparable with those of day 10 α-SMA staining in Supplementary Fig. S1.

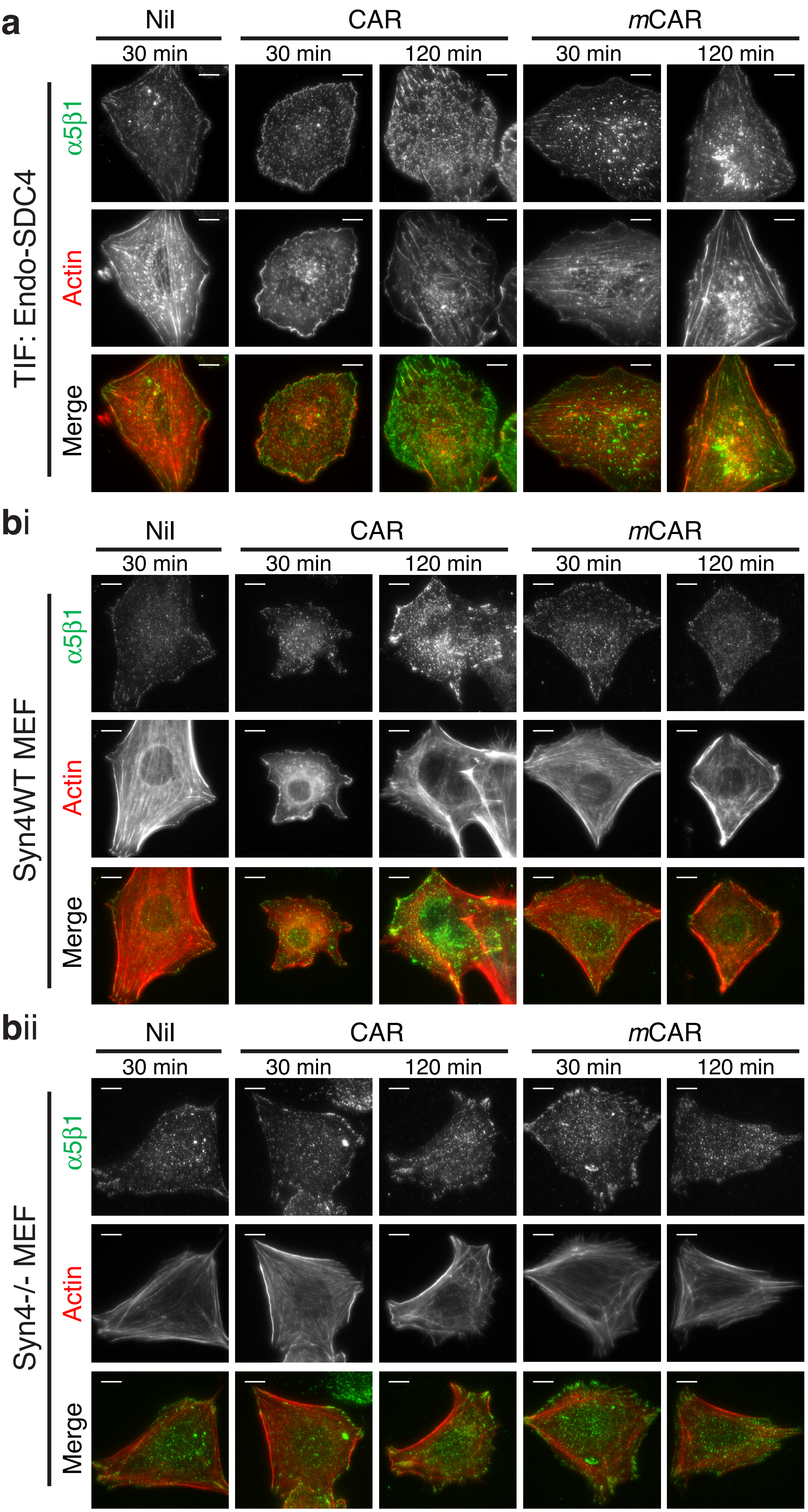
a
Nil
CAR
mCAR
30 min
30 min
120 min
30 min
120 min
TIF: Endo-SDC4
α5β1
Actin
Merge
bi
Nil
CAR
mCAR
30 min
30 min
120 min
30 min
120 min
Syn4WT MEF
α5β1
Actin
Merge
bii
Nil
CAR
mCAR
30 min
30 min
120 min
30 min
120 min
Syn4-/- MEF
α5β1
Actin
Merge

Figure S4

**Supplementary Figure S4. SDC4 regulates CAR peptide-mediated focal adhesion and cytoskeletal reorganisation**

Representative immunofluorescence micrographs of cells adherent to central cell-binding domain of fibronectin (Fn6-10/50K) for 120 mins, prior to 30- or 120-mins stimulation with 10 μg/ml CAR or *m*CAR peptides or vehicle control. **(a)** Subcellular distribution of $\alpha5\beta1$ integrin (Green) and actin (Red) in TIFs. **(b)** Subcellular distribution of $\alpha5\beta1$ integrin (Green) and actin (Red) in Syn4WT MEFs **(b**i**)** and Syn4-/- MEFs **(B**ii**)**. Scale bar = 10μm.

Figure S5

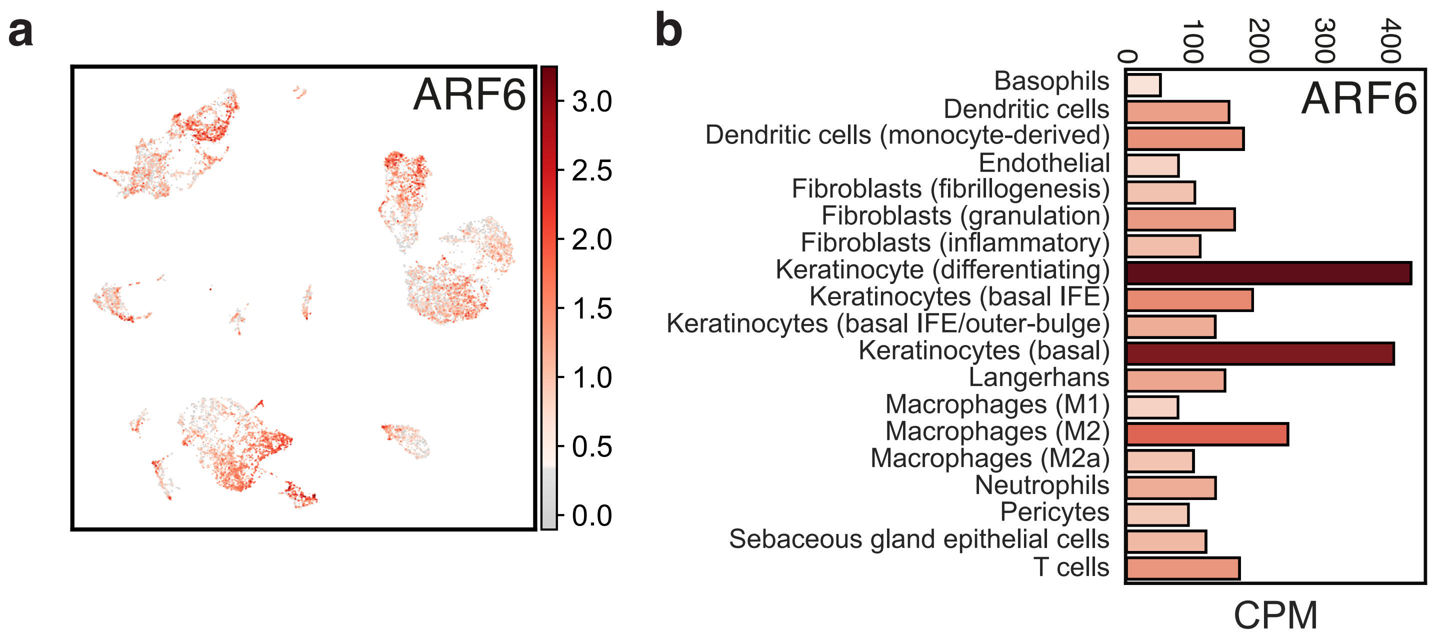
a
ARF6
3.0
2.5
2.0
1.5
1.0
0.5
0.0
b
0
100
200
300
400
ARF6
Basophils
Dendritic cells
Dendritic cells (monocyte-derived)
Endothelial
Fibroblasts (fibrillogenesis)
Fibroblasts (granulation)
Fibroblasts (inflammatory)
Keratinocyte (differentiating)
Keratinocytes (basal IFE)
Keratinocytes (basal IFE/outer-bulge)
Keratinocytes (basal)
Langerhans
Macrophages (M1)
Macrophages (M2)
Macrophages (M2a)
Neutrophils
Pericytes
Sebaceous gland epithelial cells
T cells
CPM

**Supplementary Figure S5. ARF6 is highly expressed in wound keratinocytes**

Single-cell RNA-Seq analysis of ARF6 expression in skin wounds. Single-cell transcriptomics analysis of gene expression in different cells in skin wounds[1]. scRNA-Seq data from 16,351 cells was analyzed using the SCANPY Python library[2] and clusters identified using Louvain clustering at resolution 0.75. Clusters were visualized with the UMAP model and cell types determined by literature review of highly upregulated genes in each Louvain cluster. See Figure 5g. **(a)** ARF6 expression mapped onto all cell types within UMAP model. **(b)** Barplot presenting average counts per million (CPM) for ARF6 across each identified cell type.

Figure S6

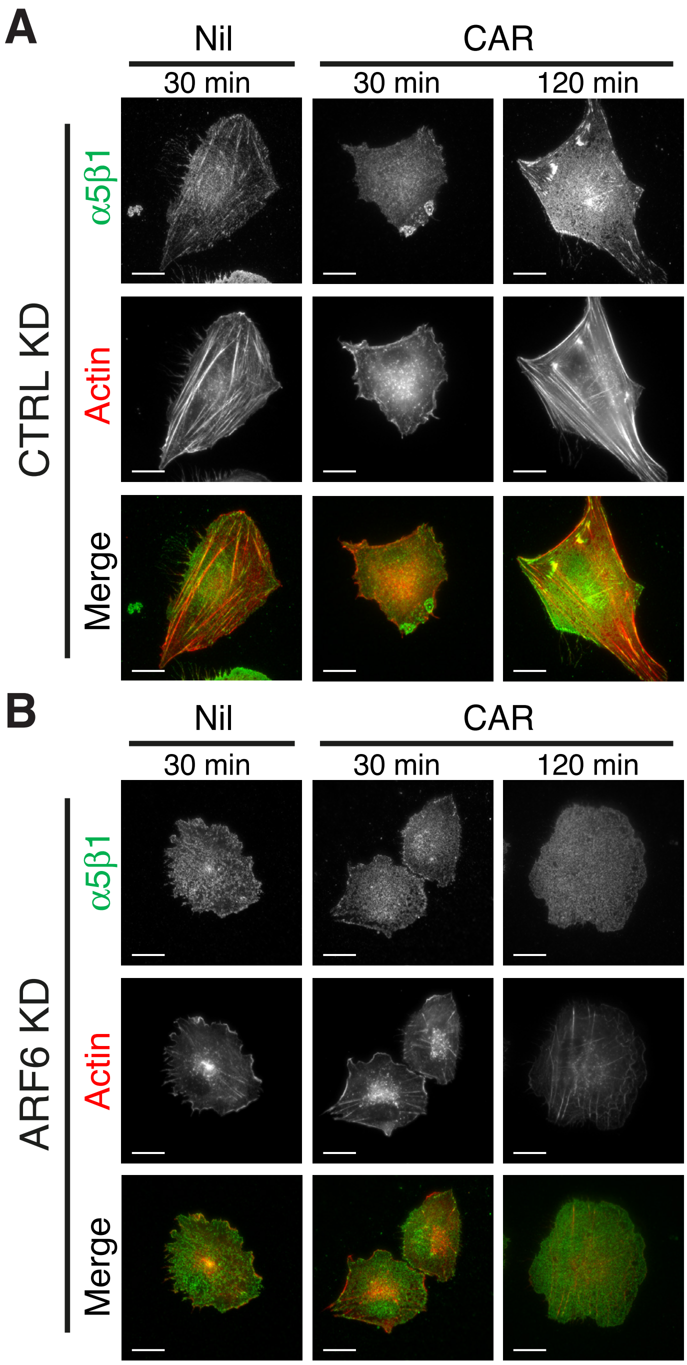
A
Nil
CAR
30 min
30 min
120 min
CTRL KD
α5β1
Actin
Merge
B
Nil
CAR
30 min
30 min
120 min
ARF6 KD
α5β1
Actin
Merge

**Supplementary Figure S6. ARF6 is required for CAR peptide-mediated focal adhesion and cytoskeletal reorganisation**

Representative immunofluorescence micrographs of **(a)** CTRL KD and **(b)** ARF6 KD TIFs adherent to central cell-binding domain of fibronectin (Fn6-10/50K) for 120 mins, prior to 30- or 120-mins stimulation with 10 µg/ml CAR or *m*CAR peptides or vehicle control. Images display subcellular distribution of $\alpha5\beta1$ integrin (Green) and actin (Red). Scale bar = 20µm.

# Figure S7

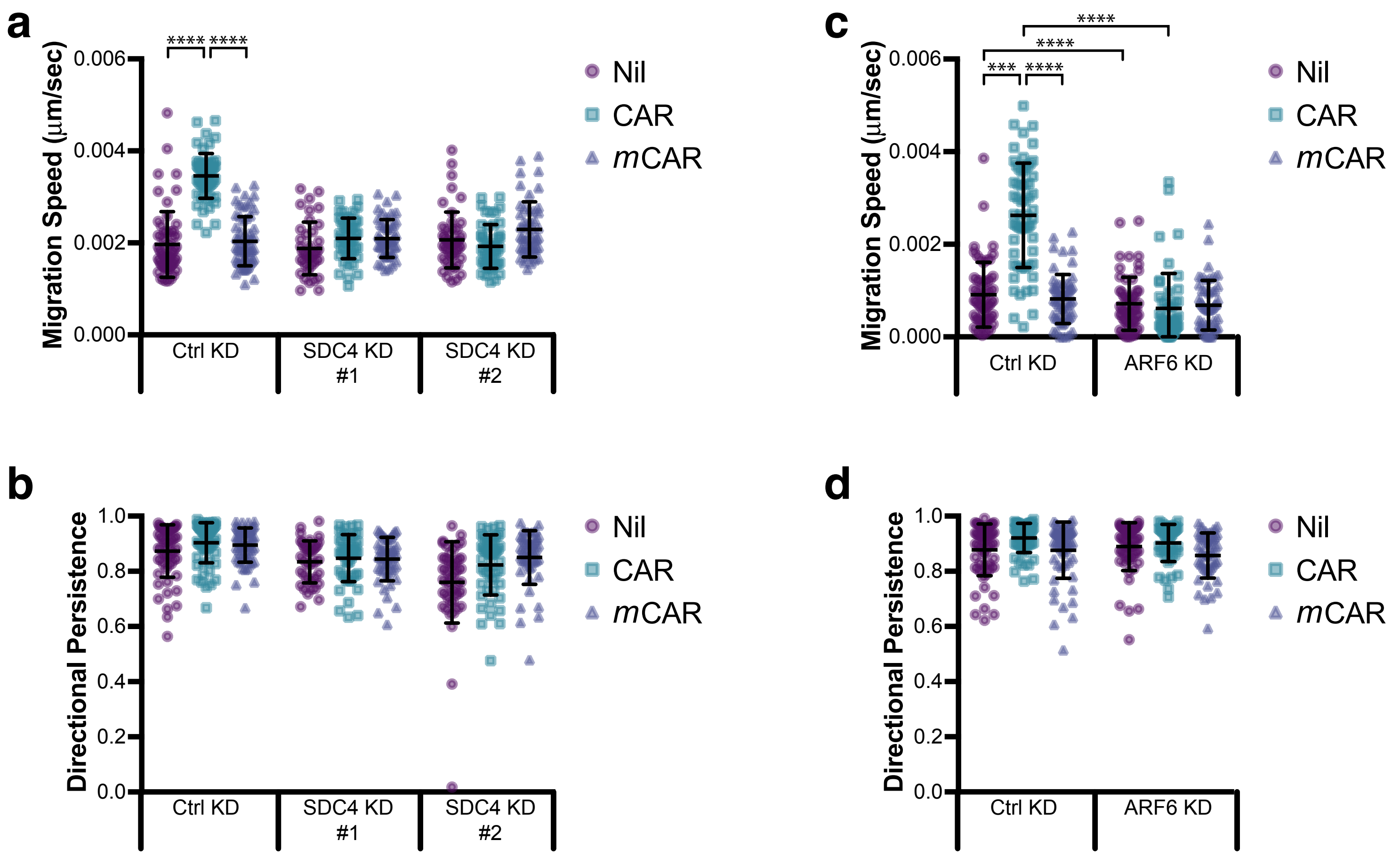

a
Migration Speed (μm/sec)
0.006
0.004
0.002
0.000
**** ****
Ctrl KD
SDC4 KD #1
SDC4 KD #2
Nil
CAR
mCAR
c
Migration Speed (μm/sec)
****
****
*** ****
Ctrl KD
ARF6 KD
b
Directional Persistence
1.0
0.8
0.6
0.4
0.2
0.0
d

**Supplementary Figure S7. CAR promotes SDC4- and ARF6-dependent keratinocyte migration**

Migration of HaCaT keratinocytes on fibronectin in scratch wound assays, in the presence or absence of 10 µg/ml CAR or *m*CAR peptide. Cells were analysed over 20 hours **(a–b)** or 17 hours **(c–d)** by time-lapse microscopy. **(a–b)** HaCaT cells transfected with control siRNA (CTRL KD), human SDC4-targeting siRNA oligo #1 (SDC4 KD #1) or human SDC4-targeting siRNA oligo #2 (SDC4 KD #2). **(a)** Mean migration speed throughout timelapse, **(b)** directional persistence throughout timelapse. Data are representative from one of four independent experiments and further data are presented in Fig. 7a–d. Values are means ± S.D. Each data point represents an individual cell. *n*=40–60 cells per condition. Statistical analyses are two-way ANOVA with Tukey's multiple comparisons test. **(a)** CTRL KD Nil vs CAR $P=1.087x10^{-12}$; CTRL KD CAR vs *m*CAR $P=1.087x10^{-12}$. **(c/d)** HaCaT cells transfected with control siRNA (CTRL KD) or human ARF6-targeting siRNA. **(c)** Mean migration speed throughout timelapse, **(d)** directional persistence throughout timelapse. Data are representative from one of four independent experiments and further data are presented in Fig. 7e–h. Values are means ± S.D. Each data point represents an individual cell. *n*=49–60 cells per condition. Source data are provided as a Source Data file. Statistical analyses are two-way ANOVA with Tukey's multiple comparisons test. **(C)** CTRL KD Nil vs CAR P=0.0002; CTRL KD CAR vs *m*CAR $P=5.689x10^{-5}$; CTRL KD Nil vs ARF6 KD Nil $P=5.26x10^{-13}$; CTRL KD CAR vs ARF6 KD CAR $P=8.276x10^{-12}$.

Figure S8

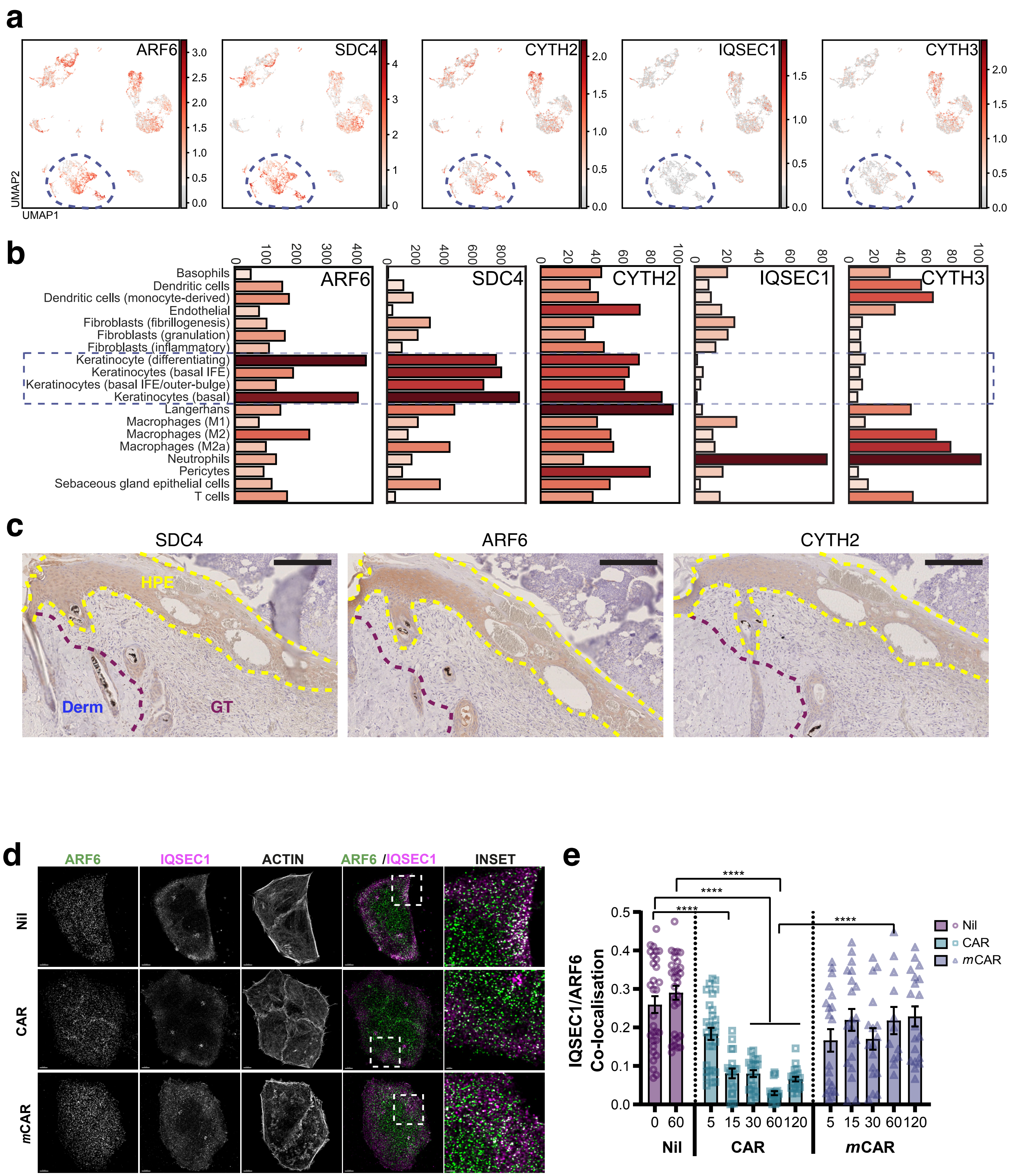

a
ARF6
SDC4
CYTH2
IQSEC1
CYTH3
UMAP2
UMAP1
b
Basophils
Dendritic cells
Dendritic cells (monocyte-derived)
Endothelial
Fibroblasts (fibrillogenesis)
Fibroblasts (granulation)
Fibroblasts (inflammatory)
Keratinocyte (differentiating)
Keratinocytes (basal IFE)
Keratinocytes (basal IFE/outer-bulge)
Keratinocytes (basal)
Langerhans
Macrophages (M1)
Macrophages (M2)
Macrophages (M2a)
Neutrophils
Pericytes
Sebaceous gland epithelial cells
T cells
c
SDC4
ARF6
CYTH2
HPE
Derm
GT
d
ARF6
IQSEC1
ACTIN
ARF6 /IQSEC1
INSET
Nil
CAR
mCAR
e
IQSEC1/ARF6 Co-localisation
Nil
CAR
mCAR
****

**Supplementary Figure S8. CYTH2 is highly expressed in wound keratinocytes alongside SDC4 and ARF6 and CAR peptide reduces colocalisation between ARF6 and IQSEC1**

**(a/b)** Single-cell RNA-Seq analysis of ARF6, SDC4, CYTH2, IQSEC1 and CYTH3 expression in skin wounds. Single-cell transcriptomics analysis of gene expression in different cells in skin wounds[1]. scRNA-Seq data from 16,351 cells was analysed using the SCANPY Python library[2] and clusters identified using Louvain clustering at resolution 0.75. Clusters were visualised with the UMAP model and cell types determined by literature review of highly upregulated genes in each Louvain cluster. See Figure 5g. **(a)** ARF6, SDC4, CYTH2, IQSEC1 and CYTH3 expression mapped onto all cell types within UMAP model. Dashed region demarcates keratinocyte subtypes. **(b)** Barplots presenting average counts per million (CPM) across each identified cell type are given for each gene. Dashed rectangle demarcates keratinocyte subtypes. SDC4 and ARF6 data are reproduced from Figure 5h/i and Supplementary Figure s4a/b, respectively, to highlight co-expression profiles. **(c)** IHC analysis of full thickness skin excision wounds. Samples were harvested from untreated mice 7 days post wounding and representative micrographs are presented of skin wound adjacent serial sections stained for SDC4, ARF6 and CYTH2. Epidermis is highlighted with yellow dashed lines. Boundary between granulation tissue and dermis is marked by red dashed lines. HPE: Hyperproliferative epidermis; GT: Granulation tissue; Derm: Dermis. Scale bar: 200 µm. **(d/e)** Quantitative analysis of IQSEC1 and ARF6 colocalisation in keratinocytes following stimulation with CAR peptide. **(d)** Representative immunofluorescence micrographs showing subcellular distribution of IQSEC1 (magenta) and ARF6 (green) in HaCaT cells following 60-mins treatment with 10 µg/ml CAR, *m*CAR or vehicle control. Dashed boxes indicate inset regions. Scale bars: 5µm (main images); 2µm (inset images). **(e)** Graph showing Pearson's coefficient of IQSEC1 and ARF6 colocalisation ± S.E.M. following 0-, 5-, 15-, 30-, 60- and 120-mins treatment with

10 µg/ml CAR or vehicle control. Each data point represents mean Pearson's coefficient of IQSEC1 and ARF6 colocalisation per cell. N=3 independent replicate experiments with 11-30 images analysed per condition. Source data are provided as a Source Data file. Kruskal-Wallis test, followed by Dunn's multiple comparisons test: Nil 0' vs CAR 15' $P=8.510x10^{-5}$; Nil 0' vs CAR 30' $P=5.970x10^{-5}$; Nil 0' vs CAR 60' $P=5.939x10^{-11}$; Nil 0' vs CAR 120' $P=1.468x10^{-6}$; Nil 60' vs CAR 60' $P=2.5x10^{-13}$; CAR 60' vs *m*CAR 60' $P=6.669x10^{-5}$.

**SUPPLEMENTARY REFERENCES**

1. Haensel, D. *et al.* Defining Epidermal Basal Cell States during Skin Homeostasis and Wound Healing Using Single-Cell Transcriptomics. *Cell Reports* **30**, 3932-3947.e6 (2020).
2. Wolf FA, Angerer P, Theis FJ. SCANPY: large-scale single-cell gene expression data analysis. *Genome Biol* **19**, 15, (2018).
3. Donaldson, J. G. & Jackson, C. L. ARF family G proteins and their regulators: roles in membrane transport, development and disease. *Nat Rev Mol Cell Biol* **12**, 362–375 (2011).
