# Supplementary material for: Systemically administered wound-homing peptide accelerates wound healing by modulating syndecan-4 function
Source: Nat Commun. 2023 Dec 6;14:8069. doi: 10.1038/s41467-023-43848-1 (PMC10700342; doi:10.1038/s41467-023-43848-1)
Supplement: Supplementary file 3 — Description of Additional Supplementary Files [file 41467_2023_43848_MOESM3_ESM.pdf]

Description of Additional Supplementary Files

Supplementary Datasets

Supplementary Data 1: ARF regulatory molecules co-immunoprecipitating with SDC4 Proteomic analysis of ARF regulatory molecules co-immunoprecipitating with human SDC4 (huSDC4) from Syn4WT, Syn4-/-, Syn4Y180E and Syn4Y180L MEFs. Spreadsheet shows known ARF regulatory proteins identified via membership of Biological Process GO Term "Regulation of ARF Protein Signal Transduction" [GO: 0032012] or reported ARF GEF or GAP activity. Orange rows and bold font indicates ARF regulatory proteins identified as co-immunoprecipitating with huSDC4 in the proteomic dataset. GEFs highlighted with green and GAPs highlighted with purple cells in "GTPase Regulation Mechanism" column. The Biological Process GO Term "Regulation of ARF Protein Signal Transduction" [GO: 0032012] is highlighted with blue text in the Gene Ontology column. "Evidence of ARF6 Activity Modulation" is based on Ref3. Blue cells highlight ARF GTPase proteins ARF6 and ARF1 not found in GO: 0032012.

Supplementary Movies

Supplementary Movie 1: CAR peptide stimulates keratinocyte migration Migration of HaCaT keratinocytes on fibronectin in scratch wound assays, in the presence or absence of 10 µg/ml CAR or mCAR peptide. Frame rate: 10 mins per frame; total time: 21 hours. See also Figure 3A–D.

Supplementary Movie 2: CAR peptide stimulates keratinocyte migration during late phase migration Migration tracks of HaCaT keratinocytes on fibronectin in scratch wound assays, in the presence or absence of 10 µg/ml CAR or mCAR peptide, during late phase migration (between 15–20 hours of 21 hour time-course). Tracking rate: 30 mins per data point; total time: 5 hours. See also Figure 3D/E.

Supplementary Movie 3: CAR promotes SDC4-dependent keratinocyte migration Migration of HaCaT keratinocytes on fibronectin in scratch wound assays, in the presence or absence of 10 µg/ml CAR or mCAR peptide, following siRNA-mediated suppression of SDC4. Frame rate: 30 mins per frame; total time: 17 hours. See also Figures 7A–D and S6A/B.

Supplementary Movie 4: CAR promotes SDC4-dependent keratinocyte migration Migration tracks of HaCaT keratinocytes on fibronectin in scratch wound assays, in the presence or absence of 10 µg/ml CAR or mCAR peptide, following siRNA-mediated suppression of SDC4, during late phase migration (between 12–17 hours of 17 hour timecourse). Tracking rate: 30 mins per data point; total time: 5 hours. See also Figures 7C/D.

Supplementary Movie 5: ARF6 regulates CAR-stimulated keratinocyte migration Migration of HaCaT keratinocytes on fibronectin in scratch wound assays, in the presence or absence of 10 µg/ml CAR or mCAR peptide, following siRNA-mediated suppression of ARF6. Frame rate: 30 mins per frame; total time: 20 hours. See also Figures 7E–H and S6C/D.

Supplementary Movie 6: ARF6 regulates CAR-stimulated keratinocyte migration Migration tracks of HaCaT keratinocytes on fibronectin in scratch wound assays, in the presence or absence of 10 µg/ml CAR or mCAR peptide, following siRNA-mediated suppression of ARF6, during late phase migration (between 15–20 hours of 20 hour timecourse). Tracking rate: 30 mins per data point; total time: 5 hours. See also Figures 7G/H.
